# Effects of Dim Light at Night on Food Intake and Body Mass in Developing Mice

**DOI:** 10.3389/fnins.2017.00294

**Published:** 2017-05-26

**Authors:** Yasmine M. Cissé, Juan Peng, Randy J. Nelson

**Affiliations:** ^1^Behavioral Neuroendocrinology Group, Department of Neuroscience, Neuroscience Research Institute, The Ohio State University Wexner Medical CenterColumbus, OH, USA; ^2^Center for Biostatistics, The Ohio State University Wexner Medical CenterColumbus, OH, USA

**Keywords:** light at night, circadian disruption, metabolism, juvenile, adolescence, early life

## Abstract

Appropriately timed light is critical for circadian organization; exposure to dim light at night (dLAN) disrupts temporal organization of endogenous biological timing. Exposure to dLAN in adult mice is associated with elevated body mass and changes in metabolism putatively driven by voluntary changes in the time of food intake. We predicted that exposure of young mice to LAN could affect adult metabolic function. At 3 weeks (Experiment 1) or 5 weeks (Experiment 2) of age, mice were either maintained in standard light-dark (DARK) cycles or exposed to nightly dLAN (5 lux). In the first two experiments, food intake and locomotor activity were assessed after 4 weeks and a glucose tolerance test was administered after 6 weeks in experimental lighting conditions. In Experiment 3, tissues were collected around the clock at 6 h intervals to investigate rhythmic hepatic clock gene expression in mice exposed to dLAN from 3 or 5 weeks of age. Male and female mice exposed to dLAN beginning at 3 weeks of age displayed similar growth rates and body mass to DARK-reared offspring, despite increasing day-time food intake. Exposure to dLAN beginning at 5 weeks of age increased body mass and daytime food intake in male, but not female, mice. Consistent with the body mass phenotype, clock gene expression was unaltered in the liver. In contrast to adults, dLAN exposure during the development of the peripheral circadian system has sex- and development-dependent effects on body mass gain.

## Introduction

Early postnatal life is a critical time for shaping both neural and physiological systems, ultimately influencing physiology, behavior, and health in adulthood (Plagemann et al., 2012). Social and physiological stressors during gestation and early postnatal development have long–term effects into adulthood (Fagundes et al., 2013; Szyf, 2013). Early life is also a critical time for shaping the circadian system (Davis and Reppert, 2001). Circadian rhythms are endogenous 24-h cycles of physiology and behavior, synchronized to the external day-night cycle by photic input to the suprachiasmatic nucleus (SCN) of the hypothalamus. Endogenous rhythmicity is maintained by a transcriptional-translational feedback loop consisting of a core set of clock genes (Mohawk et al., 2012). Although, rhythmic clock gene expression in the rodent SCN is solidified and responsive to light by the first week of postnatal life (Christ et al., 2012), peripheral tissue clock gene expression, such as the liver, does not stabilize until much later in development (Yamazaki et al., 2009).

The liver is regulated by the circadian system: 10% of the liver transcriptome and 20% of the proteome display circadian rhythms (Akhtar et al., 2002; Panda et al., 2002; Reddy et al., 2006; Miller et al., 2007; Eckel-Mahan et al., 2012). This is reflected in the robust circadian pattern in the hepatic glucose and triglyceride regulation (la Fleur et al., 2001; Rudic et al., 2004; Adamovich et al., 2014). In rats, the liver undergoes a ~4 h phase advance in peak clock gene *Per1* and *Rev-Erb*α expression from weaning into adulthood (Sládek et al., 2007). Recent studies suggest that early life lighting environment affects development of the circadian system and can have long-term effects on adult physiology and behavior (Brooks and Canal, 2013; Fonken and Nelson, 2015).

Light is the most potent signals for entraining the circadian system to the external day-night cycles, and exposure to light at night causes significant circadian system disruption (Navara and Nelson, 2007). Compared to the ~1 lux of light reflected by the moon, modern environments present an excess of light input to the circadian system at night and dysregulates clock-setting (Cinzano et al., 2001). Exposure to dim light (5 lux) at night (dLAN) increases body mass and impairs glucose tolerance in Swiss Webster mice (Fonken et al., 2010), correlated with decreased amplitudes of central and peripheral clock gene expression (Fonken et al., 2013). Circadian disruption in general, is associated with the development of obesity and metabolic syndrome (Shi et al., 2013), impaired memory (Craig and McDonald, 2008; Loh et al., 2010), and cognitive flexibility (Karatsoreos et al., 2011). However, to date these correlations have focused on the effect of light at night on adult rodents; although light at night during early life is prevalent, its effects on juveniles and adolescents remain largely unspecified.

Children are not exempt from exposure to LAN. Greater than a third of American children ages 1–7 have televisions in their bedrooms (Cespedes et al., 2014) and watching television is often a part of their children's bedtime routine (Owens et al., 1999). Exposure to dLAN during gestation or during the first 3 weeks of life alters growth trajectory post weaning and increases anxiety-like behavior in mice (Borniger et al., 2014). Juvenile and adolescent exposure to dLAN increases anxiety-like behavior and disrupts hypothalamic clock gene expression (Cissé et al., 2016). Disruption of early postnatal central and peripheral clock gene expression precede an adult obesity phenotype (Sutton et al., 2010). Critical periods for behavioral and metabolic priming occur from gestation through pre-adolescent development (Dietz, 1994), but few studies have examined this later period in development (Eiland et al., 2012; Boitard et al., 2015) and much less as it relates to circadian dysfunction. The present study will investigate the time dependent effect of LAN during early life on physiology and behavior. We hypothesized that exposure to LAN at beginning at juvenile (3 week) and adolescent (5 week) developmental epochs disrupts peripheral circadian rhythms regulating body mass and glucose processing.

## Materials and methods

### Mice

Adult male and female Swiss-Webster mice (7 weeks of age) were obtained from Charles River Laboratories to serve as breeding pairs. Mice were housed in heterosexual pairs in polypropylene cages (30 × 15 × 14 cm) at an ambient temperature of 22 ± 1°C, relative humidity of 25 ± 10%, and 14:10 h light cycle with lights on at 02:00 h and lights off at 16:00 h Eastern Time (DARK). Regular chow (Harlan Teklad 8640; Madison, WI) and filtered tap water were available *ad libitum*. Pups obtained from these pairings were housed individually after weaning. All experimental procedures were approved by The Ohio State University Institutional Animal Care and Use Committee, and animals were maintained in accordance with the recommendations of the National Institutes of Health and *The Guide for the Care and Use of Laboratory Animals*.

### Experimental design

Litter sizes were normalized to 8–10 pups resulting in a total of 67 pups. Pups were tattooed at PD0 to maintain records of pre-weaning weights. Body mass was recorded weekly. Weanlings were single housed at weaning. Mice were randomly assigned into either 14:10 DARK (~130 lux/0 lux) or dLAN (~130 lux/5 lux) conditions at either 3 weeks (Experiment 1) or 5 weeks of age (Experiment 2). Experiment 3 comprised a separate subset of mice reared in an identical manner to Experiments 1 and 2, that were killed at 4 timepoints around the clock, (Zeitgeber time [ZT])] 02:00, 8:00, 14:00, and 20:00) to assess clock genes.

#### Experiment 1. effect of juvenile exposure to light at night on body mass

Male and female offspring were reared in DARK nights until 3 weeks of age. At weaning, mice were individually housed and either maintained in DARK nights or transferred to dLAN conditions. Four weeks after onset of experimental lighting conditions, food pellets were weighed twice daily, at dark and light onset, to assess timing and quantity of food intake over 5 days. Timing of food intake was quantified as a percentage of food consumed during the light phase/total daily consumption averaged over the last 4 days of food measurement. At 9 weeks of age a glucose tolerance test was administered, as described below.

#### Experiment 2. effect of adolescent exposure to light at night on body mass

Male and female offspring were reared in standard light dark conditions until 5 weeks of age. 5-week-old mice were separated into DARK and dLAN lighting conditions as described above. Food intake and locomotor activity were measured at 9 weeks of age, after 4 weeks in lighting conditions. At 11 weeks of age mice administered a glucose tolerance test (GTT).

#### Experiment 3. early life exposure to light at night on clock gene expression

Male and female offspring were reared in standard lighting conditions until 3 and 5 weeks of age, respectively, at which point they were separated into DARK and dLAN lighting conditions as described above. After 6 weeks in their respective lighting conditions, mice were killed at 4 timepoints around the clock, (Zeitgeber time [ZT] 02:00, 08:00, 14:00, and 20:00). Mice were anesthetized with isoflurane vapors, livers were removed, weighed and flash frozen for analysis of clock gene expression using qPCR.

### Locomotor activity measurements

Spontaneous general locomotor activity was recorded in 7 mice per group using OPTO M3 animal activity monitors (Columbus Instruments; Columbus, OH, USA). Data were continuously compiled by MDI software and analyzed in order to assess presence circadian organization and quantify physical activity as a mediator of altered metabolism.

### Glucose tolerance tests (GTT)

Glucose processing was tested after 6 weeks in lighting conditions. Mice were administered an i.p glucose bolus (1.5 g/kg body mass) at ZT5 after an 18 h fast. Blood samples of ~5 μL were collected via tail vein just prior to glucose injection and again at 15, 30, 60, and 120 min following injection. Blood glucose was measured immediately with the Contour blood glucose monitoring system and corresponding test strips (Bayer Health Care, Mishakawa, IN, USA). Post GTT animals were killed by cervical dislocation.

### qPCR

Collected tissues were homogenized and total RNA extracted using Trizol (Invitrogen, Carlsbad, CA, USA). RNA quantity and quality were determined using a spectrophotometer (NanoDrop), and was reverse transcribed into cDNA using M-MLV Reverse Transcriptase enzyme (Invitrogen). Forty nanagrams of cDNA/reaction was used in subsequent PCR. Taqman Fast advanced master mix (Life Technologies) containing AmpliTaq Fast DNA polymerase was used in a 20 μL duplex reaction with one of the primer/probe pairs listed below and a primer limited primer/probe for the endogenous control eukaryotic 18s rRNA. The 2-step real-time PCR cycling conditions used were: 95°C for 20s, and 40 cycles of 95°C for 3s then 60°C for 30s. Clock gene expression of *Clock* (Mm00455950), *Bmal1* (Mm00500226), *Per1* (Mm00501813), *Cry2* (Mm01331539), and *Rev-ERB*α (Mm00520708) was assessed in liver tissue.

### Statistical analyses

Changes in body mass and glucose tolerance with time as a within subject variable were assessed using a repeated measures ANOVA. Comparisons of weight gain, serum glucose, food intake, and locomotor activity between groups were conducted using a two-way ANOVA with lighting condition and sex as between subject factors. After discovery of a sex difference, groups were analyzed by sex with lighting condition as between subject factors. Gene expression data were represented as ΔCT values due to the unequal variance in data when transformed using relative gene expression and delta-delta CT. Gene expression was assessed using a mixed model ANOVA with age of exposure, lighting condition, time of day, and sex as within subject variables and a random plate effect to control for the multiple PCR plates needed to run all samples. Unevenly distributed data were log transformed or assessed for main effects using Mann-Whitney U tests and interactions using Kruskal-Wallis tests. *Post hoc* tests of statistically significant interactions were performed using Bonferroni for repeated measures and Tukey's HSD test for two-way ANOVA. Outliers determined by Z-score analysis and removed from analysis. Analyses were completed with SPSS software (version 24.0.0) and SAS (version 9.3). A two-sided significance level of α ≤ 0.05 was used for all tests.

## Results

### Experiment 1

#### Somatic measures

**Figure 1:**
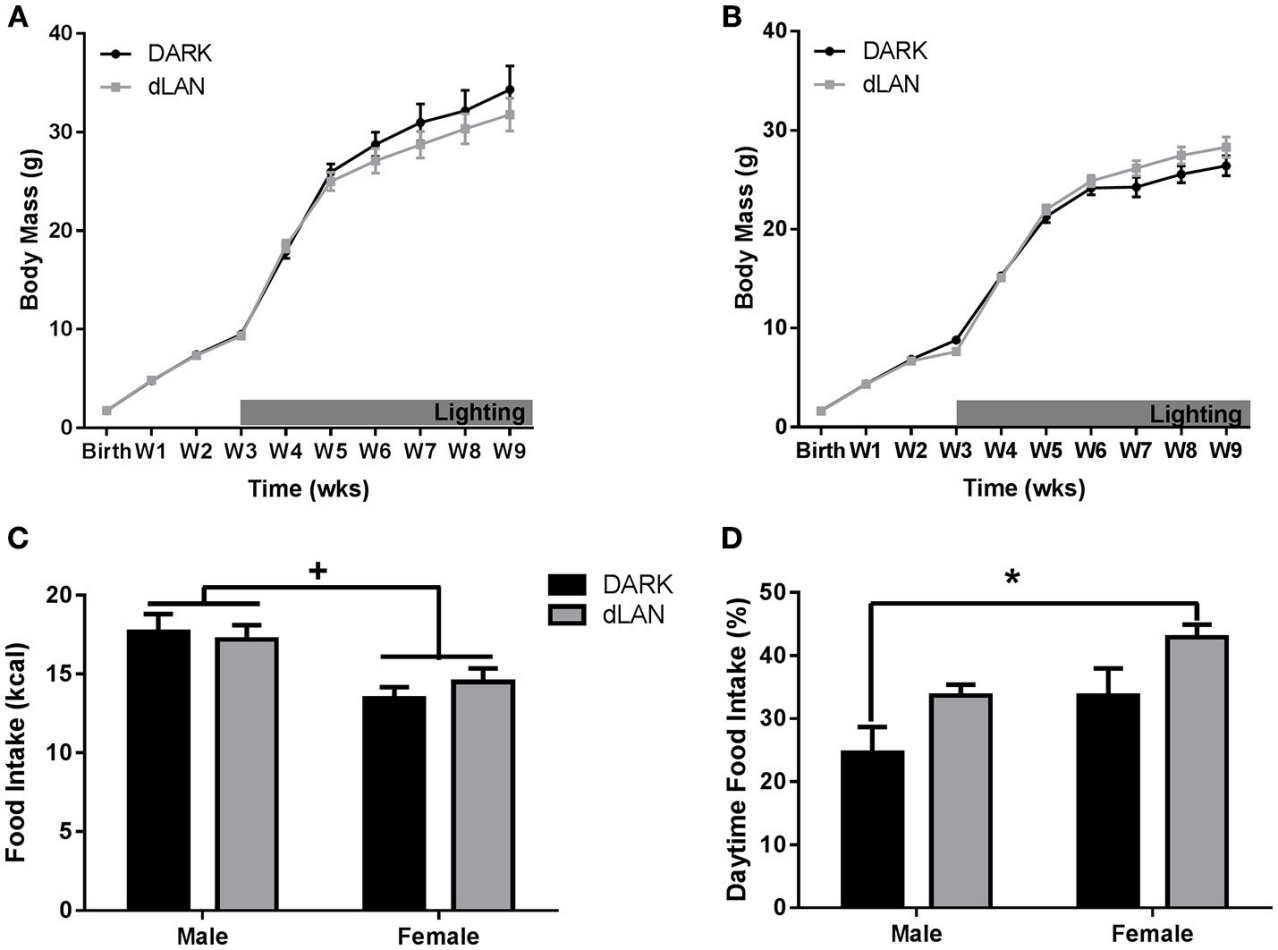
**Juvenile exposure to dLAN shifts timing of food intake without affecting body mass**. Weekly change in body mass from birth in males **(A)** and females **(B)**. Daily caloric food intake **(C)**. Relative daily food intake **(D)**. *n* = 5–8 per group per sex. Data are presented as mean ± SEM; ^*^p < 0.05 DARK vs. dLAN.

When exposed to dLAN starting at 3 weeks of age, neither males (Figure 1A) nor females (Figure 1B) gained body mass relative to mice housed in dark nights (*p* > 0.05). There was a sex difference in body mass beginning at birth with males weighing more than females [*F*_(1, 33)_ = 4.19, *p* < 0.05]. At the end of the experiment, there were no sex or light condition differences in gonadal fat mass (*p* > 0.05).

#### Food intake

Mice exposed to dLAN ate more during the daytime than mice exposed to DARK nights [*F*_(1, 33)_ = 7.74, *p* < 0.01; Figure 1D]. Daytime food intake also differed by sex [*F*_(1, 33)_ = 7.77, *p* < 0.05], such that females ate more of their food during the day than males. Daily caloric food intake did not differ between lighting conditions (*p* > 0.05; Figure 1C), but differed between sexes in that males consumed more calories than females [*F*_(1, 33)_ = 14.89, *p* < 0.01].

#### Glucose tolerance testing

Fasting glucose levels displayed a significant sex difference [*F*_(1, 14)_ = 6.349, *p* < 0.05] and in area under the curve of glucose response [*F*_(1, 14)_ = 11.14, *p* < 0.01]. Glucose tolerance also differed between sexes [*F*_(2, 34)_ = 6.92, *p* < 0.05]; thus, males and females were analyzed individually.

##### Males

**Figure 2:**
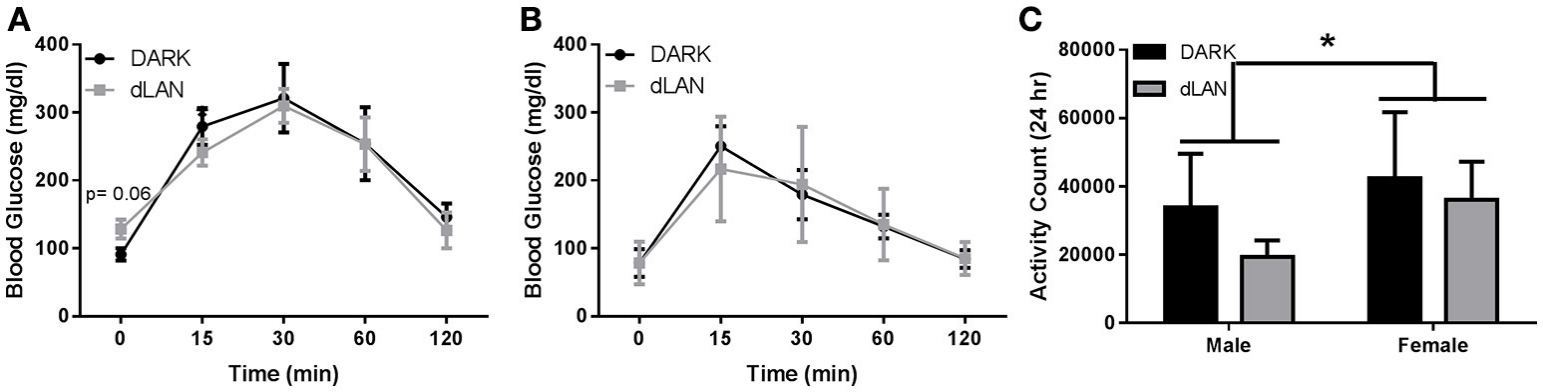
**Juvenile exposure to dLAN does not affect glucose processing**. Glucose tolerance testing in males **(A)** and females **(B)**. Blood glucose concentrations after an 18 h fast **(C)**. *n* = 4–6 per group per sex. Data are presented as mean ± SEM; ^*^p < 0.05 DARK vs. dLAN.

Baseline concentrations of glucose were marginally elevated in dLAN exposed males (*p* = 0.066; Figure 2A), but experimental lighting conditions did not alter glucose tolerance.

##### Females

Experimental lighting conditions did not alter fasted glucose levels or overall glucose tolerance in female mice (*p* > 0.05; Figure 2B).

#### Locomotor activity

Sex differences in total daily locomotor activity were observed such that females were more active than males [*F*_(1, 20)_ = 9.91, *p* < 0.05], but dLAN did not alter total activity counts (*p* > 0.05). Daytime and nighttime locomotor activity differed [*F*_(1, 20)_ = 2752.7, *p* < 0.001], but lighting condition did not affect locomotor activity in the day- or night-time (*p* > 0.05).

### Experiment 2

#### Somatic measures

**Figure 3:**
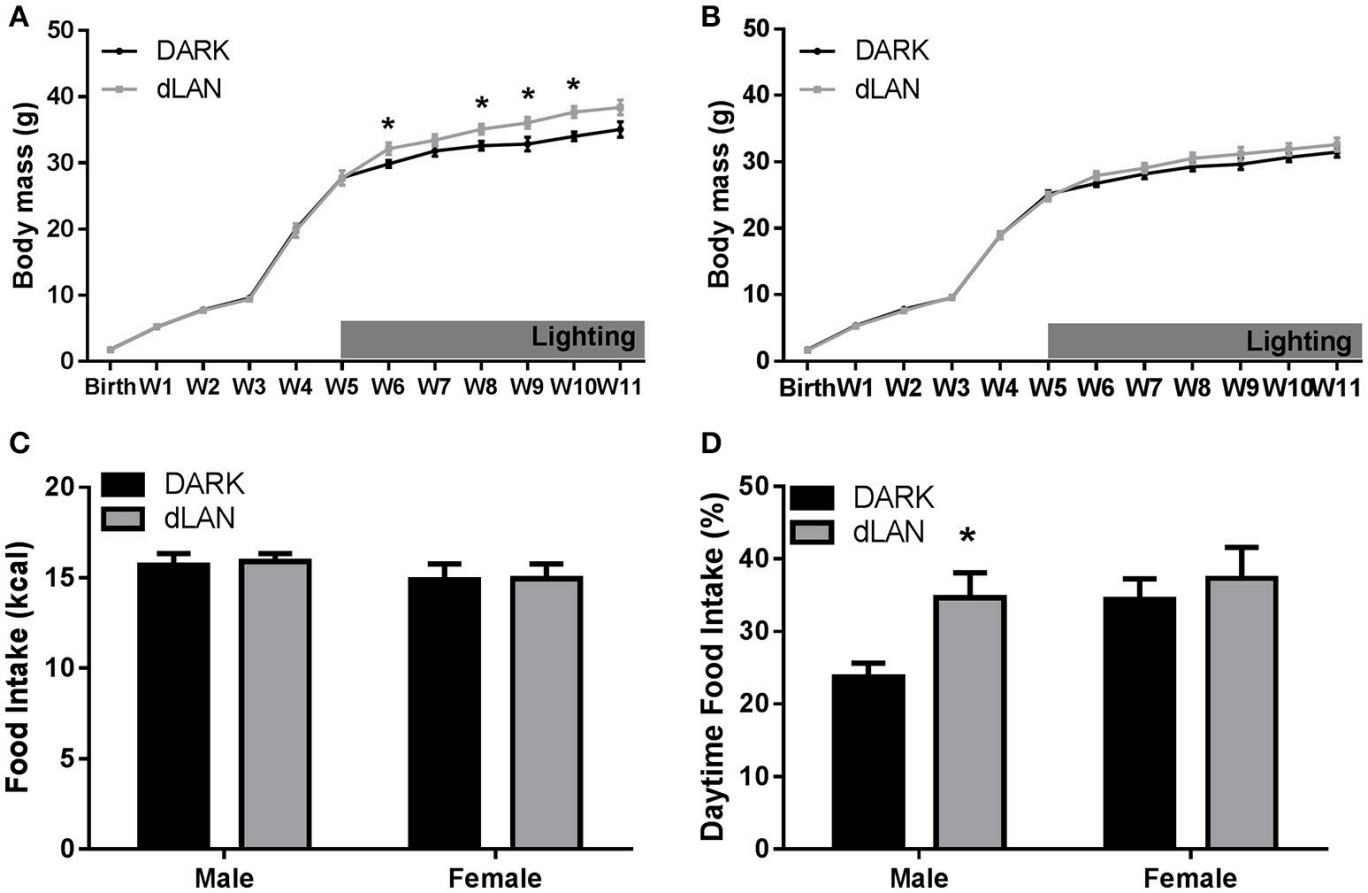
**Adolescent exposure to dLAN shifts timing of food intake without affecting body mass**. Weekly change in body mass from birth in males **(A)** and females **(B)**. Daily caloric food intake **(C)**. Relative daily food intake **(D)**. *n* = 6–8 per group per sex. Data are presented as mean ± SEM; ^*^p < 0.05 in DARK vs. dLAN.

When exposed to dLAN starting at 5 weeks of age, male mice increased body mass relative to their DARK night counterparts [*F*_(3, 38)_ = 5.58, *p* < 0.005; Figure 3A], but not females (*p* > 0.05; Figure 3B). Adolescent exposure to dLAN increased gonadal fat mass [*F*_(1, 25)_ = 5.23, *p* < 0.05]. Growth rate between males and females showed the typical sex difference such that males increased body mass more rapidly than females [*F*_(3, 79)_ = 20.23, *p* < 0.05].

#### Food intake

Males exposed to dim light during adolescence ate more during the day than males housed in dark nights [*F*_(1, 13)_ = 6.95, *p* < 0.05], whereas dLAN did not alter female daytime food consumption (*p* > 0.05; Figure 3D). Daytime food intake differed between the sexes [*F*_(1, 29)_ = 4.09, *p* = 0.05]. Total daily caloric intake did not differ between lighting conditions (*p* > 0.05; Figure 3C).

#### Glucose tolerance testing

**Figure 4:**
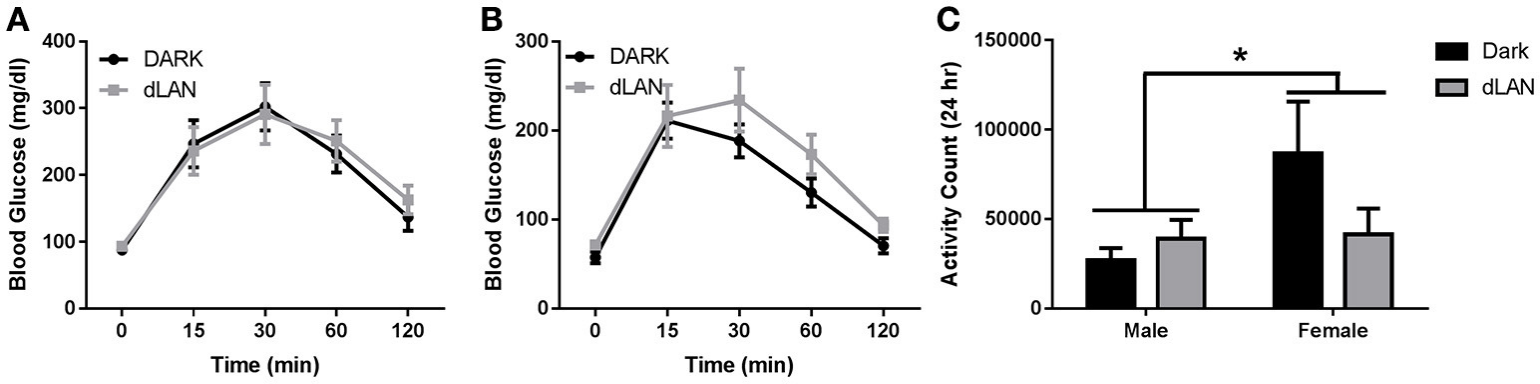
**Adolescent exposure to dLAN does not affect glucose processing. Glucose tolerance testing in males (A)** and females **(B)**. Blood glucose concentrations after an 18 h fast **(C)**. *n* = 7 per group per sex. Data are presented as mean ± SEM; ^*^p < 0.05 in DARK vs. dLAN.

There was a sex difference in area under the curve of glucose response [*F*_(1, 25)_ = 8.02, *p* < 0.01]. Exposure to dLAN during adolescence did not alter fasted glucose levels or overall glucose tolerance results in male (Figure 4A) or female (Figure 4B) mice (*p* > 0.05).

#### Locomotor activity

Total daily locomotor activity displayed a significant sex difference such that females were more active than males [*F*_(1, 20)_ = 15.48, *p* < 0.05], but dLAN did not alter total daily activity counts (*p* > 0.05). Daytime and nighttime activity significantly differed [*F*_(1, 20)_ = 2752.7, *p* < 0.001], but lighting condition did not alter diel patterns in locomotor activity (*p* > 0.05).

### Experiment 3

**Figure 5:**
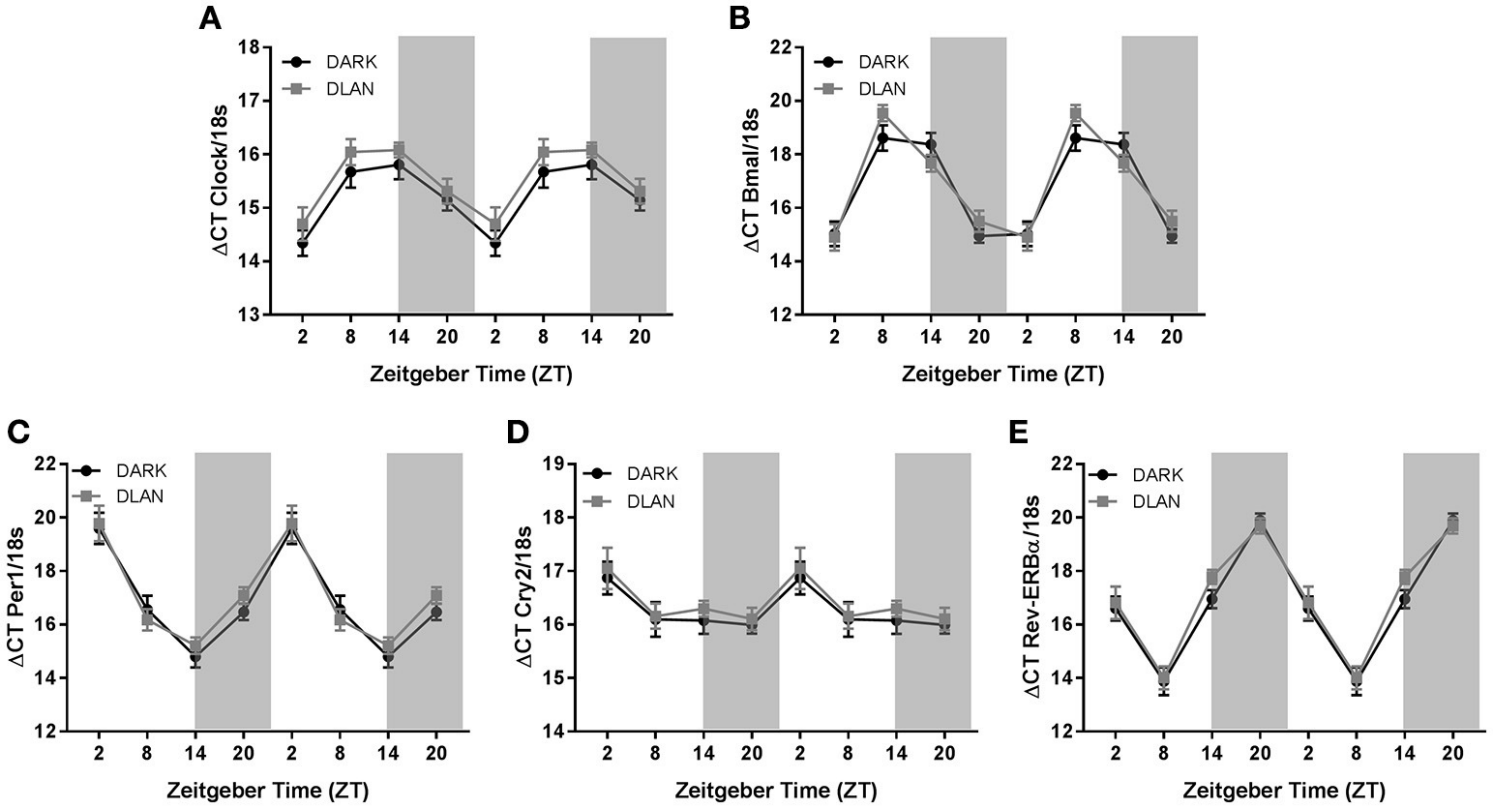
**Juvenile and adolescent exposure to dLAN does not affect hepatic clock gene expression**. Data are presented as delta CT values for hepatic *Clock* **(A)**, *Bmal1* **(B)**, *Per1* **(C)**, *Cry2* **(D)**, *Rev-ERB*α **(E)** expression and therefore appear as an inverse pattern to gene expression. *n* = 7–13 per group per sex. Data are presented as mean ΔCT ± SEM; +*p* < 0.05 time of day.

Gene expression of *Clock, Bmal1, Per1, Cry2*, and *Rev-ERB*α displayed a circadian rhythm in the liver (*p* < 0.001 in all cases; Figure 5). However, dLAN did not alter parameters of clock gene expression in the liver (*p* > 0.05).

## Discussion

The goal of this study was to evaluate the effect of dLAN in juvenile and adolescent mice on body mass and glucose metabolism. We predicted that dLAN would increase body mass, impair glucose tolerance, and alter hepatic clock gene expression. In contrast to our hypothesis, juvenile exposure to dLAN did not increase body mass or alter glucose processing despite increasing the timing of food intake to daytime in both males and females (Figures 1, 2). Adolescent male mice exposed to dLAN had a modest increase in body mass and daytime food intake, with no accompanying deficits in glucose processing (Figures 3, 4). Body mass and glucose tolerance results were unaffected by dLAN in adolescent female mice. Hepatic clock genes remained rhythmic in all mice exposed to dLAN, and expression profiles of these clock genes not differ between sexes, lighting condition, or age of the onset of dLAN exposure (Figure 5).

Exposure to dLAN increases body mass in adult male and female Swiss Webster mice, C3H/HeNHsd mice, and Siberian hamsters, but not Wistar rats (Fonken et al., 2013; Fonken and Nelson, 2013; Aubrecht et al., 2015; Stenvers et al., 2016; Cisse et al., 2017). Contrary to previous findings in adult mice, early life exposure to dLAN has little to no effect on body mass (Figures 1, 3). In line with previous reports, juvenile and adolescent mice exposed to dLAN increased daytime food intake (Figures 2, 4). Of note, the approximately 30% and 40% increase in daytime food intake exhibited by juveniles and adolescent males, respectively, is modest relative to the nearly 60% in daytime food intake observed in adults exposed to dLAN (Fonken et al., 2010). Weanlings undergo a dramatic change in time of feeding from consuming the majority of their food during the day when the dam is on the nest to eating during the night as adults (Levin and Stern, 1975). Mice exposed to dim light at 3 weeks of age may not be as disrupted by daytime feeding as adult mice with solid nighttime feeding patterns. At 5 weeks of age, the intermediary body mass gain exhibited by males suggests a progression toward the adult phenotype while maintaining some metabolic flexibility. The developmental timing of dLAN exposure, specifically when animals are developing adult rhythms in food intake, may contribute to the minimal effects on body mass in response to dLAN.

Although central circadian rhythms are established long before weaning, peripheral clocks continue to undergo rearrangements until 2 months of age (Sumová et al., 2006; Sládek et al., 2007). The liver acts as an integrator of circadian rhythms and metabolic homeostasis (Vollmers et al., 2009; Bass and Takahashi, 2010). Disruption of hepatic clock gene function impairs glucose and lipid metabolism (Lamia et al., 2008; Zhang et al., 2010; Bugge et al., 2012; Husse et al., 2012). Light at night dampens rhythmic clock gene expression in the liver, specifically *Bmal1, Per1* and *2*, and *Cry1* (Fonken et al., 2013). These animals also impair glucose tolerance in response to dLAN. Contrary to adult data, juvenile and adolescent dLAN exposed animals do not exhibit deficits in glucose processing (Figures 2, 4). These data in pre-adult mice are supported by the lack of disruption in hepatic clock gene expression (Figure 5). The liver clock is responsive to feeding cues (Stokkan et al., 2001; Vollmers et al., 2009; Adamovich et al., 2014) and restricting feeding to the active phase restores body mass and metabolic homeostasis in adult mice that have been fed a high fat diet or exposed to dLAN (Fonken et al., 2010; Hatori et al., 2012). Therefore, the moderate increases in daytime food intake may not be sufficient to alter hepatic clock gene expression and alter glucose processing.

Adult exposure to dLAN increases body mass in males, as well as females (Aubrecht et al., 2015). The results of both juvenile and adolescent females differ from adult data; female mice exposed during early life did not increase body mass higher than females exposed to dark nights. Juvenile (3 week) female mice exposed to dLAN increased daytime food intake in a comparable manner to males, but neither males nor females exhibited weight gain (Figure 1). Adolescent female mice on the other hand did not increase daytime food intake in response to dLAN despite daytime food intake being comparable to adolescent males exposed to dLAN (Figure 3). Females increased spontaneous locomotor activity relative to males, which may account for the lack of weight gain.

Overall, these results indicate that juvenile mice and adolescent female mice do not respond to dLAN by altering body mass as observed in adult mice. These results may reflect the shift from daytime nursing to post-weaning nighttime feeding on metabolism. Future studies should address the effect of timed feeding on animals exposed to dLAN in early life. If daytime feeding is responsible for hepatic clock gene disruption, then restricting feeding to the daytime should establish whether attenuated weight gain is due to reduced daytime food intake or other mechanisms of metabolic flexibility during early life.

## Ethics statement

This study was carried out in accordance with the recommendations in the Guide for the Care and Use of Laboratory Animals of the National Institutes of Health. The protocol was approved by the Ohio State University Institutional Animal Care and Use Committee (IACUC).

## Author contributions

YMC and RJN designed the experiment. YMC carried out the experiments. YMC and JP performed the statistical analyses. YMC, JP, and RJN wrote the manuscript.

### Conflict of interest statement

The authors declare that the research was conducted in the absence of any commercial or financial relationships that could be construed as a potential conflict of interest.
